# Prognostic value of CT-derived fractional flow reserve and fat attenuation index in patients with suspected coronary artery disease: a sex-disaggregated analyses

**DOI:** 10.1186/s12872-023-03650-9

**Published:** 2023-12-13

**Authors:** Yang Yu, Jieli Kou, Fuqian Guo, Dan Zhang, Tong Pan, Yicheng Chen, Wenjun Bao, Yuhan Sun, Haowen Zhang, Caiying Li

**Affiliations:** 1 Department of Medical Imaging, The Second Hospital of Hebei Medical University, Shijiazhuang, China; 2 Department of Medical Imaging, Cangzhou People’s Hospital, Cangzhou, China

**Keywords:** Coronary artery disease, CT-derived fractional flow reserve, Fat attenuation index, Prognosis, Major adverse cardiovascular events

## Abstract

**Background:**

There are sex differences in many risk factors associated with coronary artery disease (CAD). CT-derived fractional flow reserve (CT-FFR) and fat attenuation index (FAI) have been shown to independently predict cardiovascular events. We aimed to examine the impact of sex on the prognostic value of CT-FFR and FAI in suspected CAD patients, and to examine the incremental prognostic value of FAI over CT-FFR in both sex.

**Methods:**

A total of 1334 consecutive suspected CAD subjects who underwent coronary computed tomographic angiography (CCTA) were retrospectively collected. We divided the patients into males and females and calculated CT-FFR and FAI data from CCTA images. Kaplan-Meier analysis was used to assess the risk of major adverse cardiovascular events (MACE) stratified by CT-FFR and FAI in both sex. Cox regression models were used to assess the incremental prognostic value of FAI by adding the variable to a model that included CT-FFR and clinical variables.

**Results:**

During a median follow-up of 2.08 years, 212 patients had MACE. CT-FFR ≤ 0.80 was significantly associated with MACE in both sex. FAI value of left anterior descending artery (FAI[LAD]) and FAI value of left circumflex (FAI[LCX]) ≥ 70.1 were significantly associated with MACE in females. FAI[LCX] added incremental prognostic value over clinical and CT-FFR variables in females, with hazard ratio (HR) 3.230 (1.982–5.265, P = 0.000), Harrel’s C 0.669 (P < 0.001), net reclassification improvement (NRI) 0.161 (0.073–0.260, P < 0.001), and integrated discrimination index (IDI) 0.036 (0.008–0.090, P = 0.010). FAI[LAD] did not enhance risk prediction in females (Harrel’s C 0.643, P = 0.054; NRI 0.041, P = 0.189; IDI 0.005, P = 0.259). The decision curve analysis demonstrated that the model including FAI[LCX] resulted in the highest net benefit.

**Conclusions:**

In suspected CAD patients, the prognostic value of CT-FFR is not significantly biased by sex. The prognostic value of FAI[LAD] and FAI[LCX] were significantly associated with MACE in females, but not males. FAI[LCX], not FAI[LAD], added incremental prognostic value over CT-FFR and might enhance CT-FFR risk stratification in females.

Coronary artery disease (CAD) is the predominant cause of death globally. Of concern, despite adherence to current prevention therapies, patients with CAD remain at high risk for major adverse cardiovascular events (MACE) [1, 2]. Thus, predicting MACE is fundamental in identifying high-risk patients to avoid unnecessary health costs, recommending proper preventative treatment, and improving mortality.

Historically, CAD was viewed as a disease of males. Nowadays, CAD is also widely recognized to be a disease of females [3]. However, the importance of CAD in females may be underestimated due to historical biases against male CAD models and higher age-specific rates in males. In fact, there are sex differences in many risk factors associated with CAD. Mark et al. indicate that sex disaggregation should be the norm in CAD research [4]. Recently, perivascular fat attenuation index (FAI) and CT-derived fractional flow reserve (CT-FFR) have been proposed as tools for assessing coronary inflammation and hemodynamic stenosis. Inflammation impacts all phases of atherosclerosis and is associated with cardiac mortality [5]. Hemodynamic stenosis detected by CT-FFR is relative to risk assessment and clinical decision-making [6]. CT-FFR and FAI with superior functional testing and noninvasive modality have shown significant prognostic performance in known or suspected CAD patients [7, 8]. In particular, females tend to have smaller coronary arteries and more microvascular disease that could affect CT-FFR calculations [9]. Susan et al. further reported that sex differences impact the prognostic value of FAI in known or suspected CAD [10]. Additionally, the added prognostic value of CT-FFR or FAI over the clinical risk factors and conventional coronary computed tomographic angiography (CCTA) has been well demonstrated in previous studies [10, 11]. However, the sex-influenced prognostic value of CT-FFR and FAI in suspected CAD patients has rarely been demonstrated. In addition, it remains unknown whether sex influences the added prognostic value of FAI over CT-FFR.

Hence, the aim of our study was to evaluate the prognostic value of CT-FFR and FAI in suspected CAD patients disaggregated by sex, and to evaluate the incremental prognostic value of FAI over CT-FFR in both sex.

## Methods

### Study population

This is a single-center retrospective study. The study adheres to STROBE guidelines [12]. We initially analyzed 1485 consecutive individuals who underwent clinically indicated CCTA for evaluation of suspected CAD in The Second Hospital of Hebei Medical University between Dec 2019 to Jun 2021. Patients who aged 18 or older were included. The exclusion criteria were as follows: (1) patients younger than 18 years (n = 5) and repeated CCTA examination in the database (n = 26); (2) heart failure or atrial fibrillation (n = 13); (3) patients with congenital heart disease (n = 14); (4) patients with known CAD (prior myocardial infarction (MI) and/or revascularization, n = 46); (5) poor image quality or unavailable CCTA data (n = 15), and (6) lost follow-up (n = 32). Finally, a total of 1334 patients were included in the study. Baseline clinical and imaging data of both sex was examined overall from electronic medical records of the hospital and patient phone calls. The hospital ethics committee approved this study (Ethics approval number: 2023-R573). The need for informed consent was waived.

### CCTA techniques

Patients were scanned with Phillips 256-slice CT (Brilliance iCT, Philips Healthcare, Amsterdam, Netherlands). Oral Metoprolol was administered in patients with heart rate > 70 beats/min before the scan. Sublingual nitroglycerin was administered to all patients unless contraindicated. Mean heart rate was 65.5 ± 10.7 bpm.

During image acquisition, 60–80mL of iohexol (350 mg/mL, 1.0 mL/kg) was injected at a flow rate of 5–6 mL/s, followed by a 50-ml saline flush. The following scanning parameters were used: tube current was 280–370 mAs, tube voltage was 120 kV, detector collimation was 128 × 0.625 mm, layer thickness was 2.5 mm, table speed was 183 mm/second, the pitch was 0.915, gantry rotation speed was 0.4 s/rotation.

### CT-FFR and FAI

**Fig. 1:**
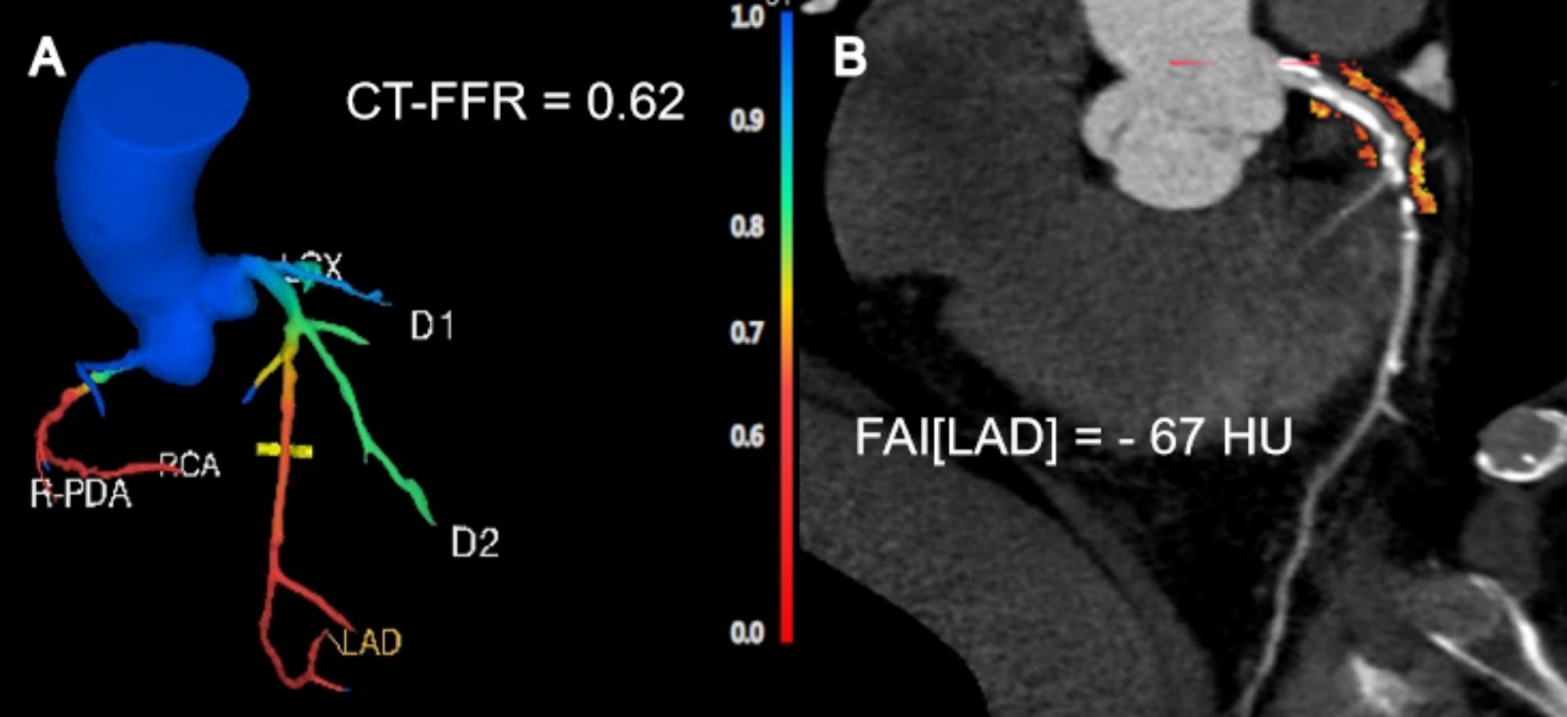
A representative case of CT-FFR and FAI assessment. (**A**) Three-dimensional color-coded mesh reveals hemodynamically significant lesions in the LAD, RCA, and PDA (CT-FFR = 0.62, CT-FFR ≤ 0.80 is considered positive). (**B**) Perivascular FAI phenotyping from CCTA in the proximal 0–40 mm of the LAD (FAI[LAD] = − 67 HU, FAI ≥ 70.1 is considered positive). CT-FFR, CT-derived fractional flow reserve; FAI, fat attenuation index; LAD, left anterior descending artery; RCA, right coronary artery; PDA, posterior descending artery; CCTA, coronary computed tomography angiography

Two radiologists (Y.Y. and Y.C. with 7 and 3 years of experience in cardiac imaging diagnosis, respectively), blinded to the results of CCTA, evaluated all CCTA images on Philips iCT EBW 4.5 post-processing workstation (Philips Healthcare, Amsterdam, Netherlands). CT-FFR and FAI were analyzed from CCTA images by two observers (Y.Y. and Y.C., respectively) using Shukun software (CT-FFR V1.7, FAI V1.2, ShuKun Technology Co., Ltd., Beijing, China). With this software, CT-FFR values were calculated for each vessel by the deep learning algorithm, 2–4 cm distal to a focal coronary lesion. For the prognostic analysis, the lowest CT-FFR value of each patient was used. The CT-FFR value was provided throughout the coronary arterial tree (Fig. 1A). To measure the perivascular FAI, we analyzed the proximal 40 mm of each vessel. Hence, the proximal 10–50 mm of the right coronary artery (RCA) and proximal 40 mm of left anterior descending artery (LAD) and the left circumflex (LCX) starting at their origin were analyzed. The FAI was defined as the mean CT attenuation value of peri-coronary adipose tissue of the traced 40 mm segment by the crude analysis. Representative images of FAI analysis were shown in (Fig. 1B). The cutoff point for perivascular FAI was − 70.1 [7].

### Follow up

All patients were followed for a median of 2.08 (1.75–2.50) years for the first occurrence of MACE, which was a composite of all-cause death, nonfatal MI, unplanned revascularization (more than 90 days after CCTA), stroke, or rehospitalisation for heart failure. MI was defined as the 4th universal definition of myocardial infarction [13]. The outcomes were obtained by telephone contact and review of medical records. The deadline date of follow-up was August 31, 2022.

### Statistical analysis

Continuous variables were expressed as mean ± standard deviation (SD) or medians (interquartile range) and categorical data as percentage. Categorical variables were compared with chi-square test or Fisher test. Continuous variables were compared with Mann-Whitney U test. The Kaplan-Meier analysis and log-rank test were performed to assess MACE rates stratified by FAI and CT-FFR. Independent predictors of MACE were analyzed with Cox regression analysis. Any risk factor that had statistically significant (*P* < 0.05) on univariable analysis was selected for multivariable modeling. Multivariable Cox regression models were used to determine the added prognostic role of FAI to CT-FFR and clinical predictors. Relative risks were expressed as multivariable-adjusted hazard ratios (HR) with 95% confidence intervals (CI). Global Chi-square value and Harrel’s C-statistic were used to assess model performance. Continuous net reclassification improvement (NRI) and absolute integrated discrimination improvement (IDI) were used to assess the risk-stratification and discrimination of models [14, 15]. Finally, Decision curve analysis [16] was obtained to compare the present model’s (model 2 and model 3) net benefit to model 1. *P* value < 0.05 was considered statistically significant. Statistical analyses were done with SPSS software (version 25.0) and R software (version 4.0.2).

## Results

### Patient characteristics

Baseline characteristics of all 1334 patients stratified by sex are depicted in Table 1. In all patients, males were significantly younger than females. In the males, the rate of smoking history, FAI[LAD] ≥ −70.1, FAI[LCX] ≥ −70.1, CT-FFR ≤ 0.80 were higher than those in the females. Females showed significantly higher CT-FFR and lower FAI[LAD]/[LCX]/[RCA] than males.

**Table 1:** Baseline characteristics in patients

	All (N = 1334)	Males (N = 704)	Females (N = 630)		*P* value
Age, years, mean ± SD	61.0 ± 10.1	59.3 ± 10.9		62.9 ± 8.8	0.000
BMI, (kg/m2), IQR	25.5 (23.5–27.8)	25.7 (23.9–27.8)		25.4 (23.4–27.6)	0.062
Hypertension, (%)	847 (63.5)	445 (63.2)		402 (63.8)	0.820
Diabetes mellitus, (%)	430 (32.2)	213 (30.3)		217 (34.4)	0.102
Hypercholesterolemia, (%)	278(20.8)	134 (19.0)		144 (22.9)	0.086
Smoking history, (%)	318 (23.8)	304 (43.2)		14 (2.2)	0.000
Family history of CAD, (%)	15 (1.1)	10 (1.4)		5 (0.8)	0.278
Aspirin/Clopidogrel, (%)	1143 (85.7)	613 (87.1)		530 (84.1)	0.125
Beta-blocker, (%)	692 (51.9)	350 (49.7)		342 (54.3)	0.095
CCB, (%)	298 (22.3)	156 (22.2)		142 (22.5)	0.868
ACEI, (%)	131 (9.8)	69 (9.8)		62 (9.8)	0.980
Statin, (%)	1203 (90.2)	628 (89.2)		575 (91.3)	0.206
Right Dominance	1213 (90.9)	630 (89.5)		583 (92.5)	0.053
left Dominance	121 (9.1)	74 (10.5)		47 (7.5)	0.053
Obstructive CAD, (%)	580 (43.5)	361 (51.3)		219 (34.8)	0.000
Maximum Severe (≥ 70%)	337 (25.3)	195 (27.7)		142(22.5)	0.030
**Lesion Location**					
Left main to LAD	283 (21.2)	181 (25.7)		102 (16.2)	0.000
LCX	224 (16.8)	135 (19.2)		89 (14.1)	0.014
RCA	173 (13.0)	95 (13.5)		78 (12.4)	0.546
FAI[LAD] (HU), IQR	-84 (-89–79)	-82 (-88–77)		-85 (-90–80)	0.000
FAI[LCX] (HU), IQR	-81 (-86–75)	-78 (-83–74)		-83 (-88–78)	0.000
FAI[RCA] (HU), IQR	-87 (-92–81)	-84 (-90–79)		-89 (-94–83)	0.000
**FAI ≥ −70.1, (%)**					
FAI[LAD] ≥ −70.1, (%)	83 (6.2)	53 (7.5)		30 (4.8)	0.037
FAI[LCX] ≥ −70.1, (%)	163 (12.2)	107(15.2)		56 (8.9)	0.000
FAI[RCA] ≥ −70.1, (%)	73 (5.5)	42 (6.0)		31 (4.9)	0.402
CT-FFR, IQR	0.88 (0.74–0.93)	0.84 (0.70–0.92)		0.90 (0.79–0.93)	0.000
CT-FFR ≤ 0.80, (%)	494 (37.0)	321 (45.6)		173 (27.5)	0.000
Cardiac catheterization, (%)	290 (21.7)	171 (24.3)		119 (18.9)	0.017
MACE, (%)	212 (15.9)	120 (17.0)		92 (14.6)	0.223
All-cause death, (%)	20 (1.5)	12 (1.7)		8 (1.3)	0.514
Nonfatal MI, (%)	129 (9.7)	72 (10.2)		57 (9.0)	0.467
Unplaned revascularzation, (%)	33 (2.5)	18 (2.6)		15 (2.4)	0.836
Stroke, (%)	12 (0.9)	7 (1.0)		5 (0.8)	0.698
Rehospitalisation for heart failure, (%)	18 (1.3)	11 (1.6)		7 (1.1)	0.476

Data are presented as mean ± SD, n (%) or median (interquartile range)

BMI, body mass index; CAD, coronary artery disease; CCB, Calcium channel blocker; ACEI, angiotensin-converting enzyme inhibitor; FAI, fat attenuation index; LAD, left anterior descending artery; LCX, left circumflex; RCA, right coronary artery; CT-FFR, CT-derived fractional flow reserve; MACE; major adverse cardiac events

### Clinical endpoints and predictors of events

**Fig. 2:**
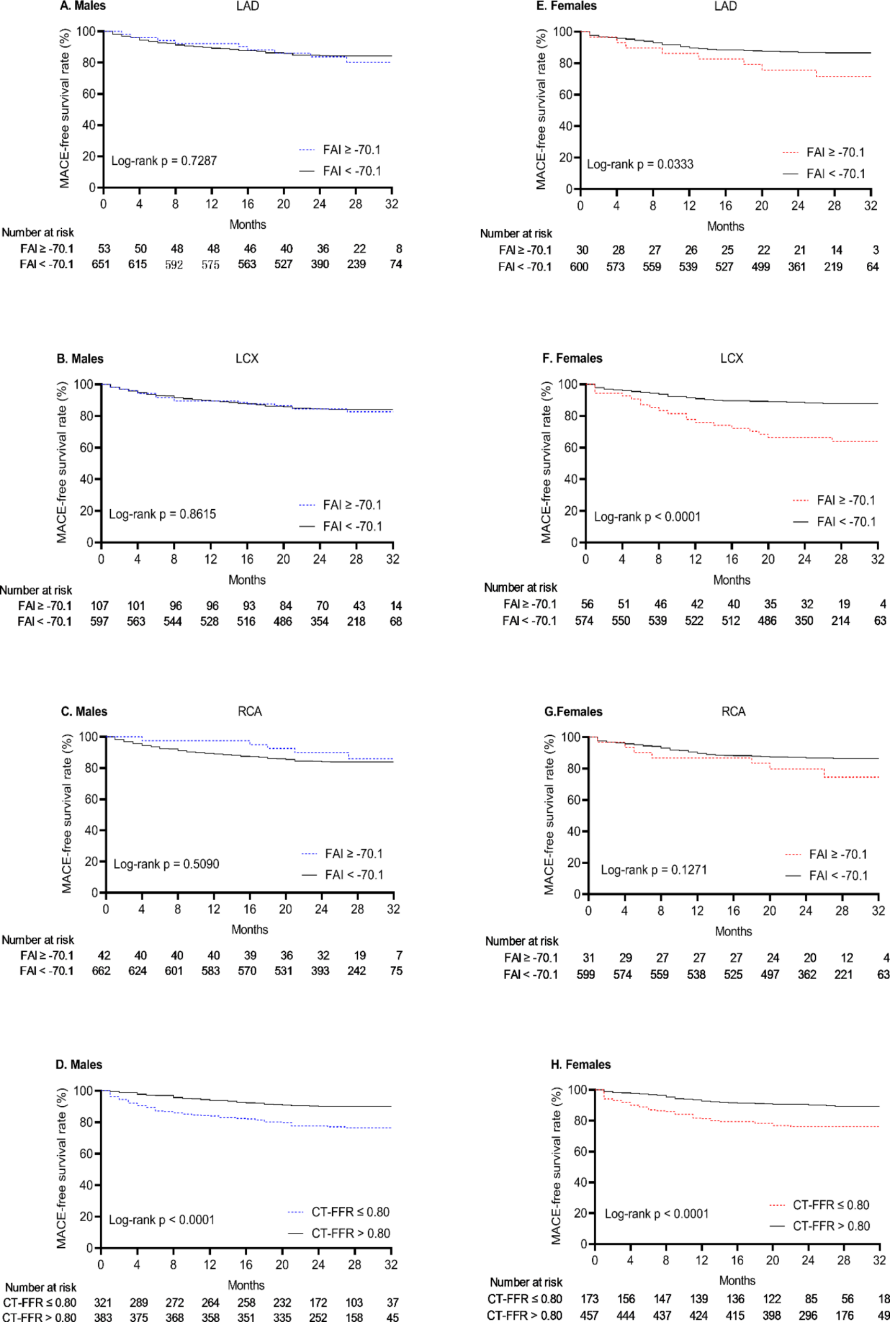
Kaplan-Meier curves showing MACE-free survival rate in males and females classified according to CT-FFR and FAI. (**A**) In males classified by FAI[LAD]. (**B**) In males classified by FAI[LCX]. (**C**) In males classified by FAI[RCA]. (**D**) In males classified by CT-FFR. (**E**) In females classified by FAI[LAD]. (**F**) In females classified by FAI[LCX]. (**G**) In females classified by FAI[RCA]. (**H**) In females classified by CT-FFR. MACE, major adverse cardiac events; CT-FFR, CT-derived fractional flow reserve; FAI, fat attenuation index; LAD, left anterior descending; LCX, left circumflex; RCA, right coronary artery

During the median follow-up of 2.08 years, 212 patients experienced at least one MACE (Table 1), there was no significant sex difference in the rate of MACE. In males, CT-FFR ≤ 0.80 was associated with the increased rate of MACE (log-rank *P* < 0.0001; Fig. 2D), while FAI[LAD]/[LCX]/[RCA] ≥ 70.1 was not associated with MACE (Fig. 2A–C). In females, similar trends of CT-FFR were observed (log-rank *P* < 0.0001; Fig. 2H). FAI[LAD] and FAI[LCX] were associated with MACE (log-rank *P* = 0.0333 and < 0.0001, respectively; Fig. 2E, F) except FAI[RCA] (log-rank *P* = 0.1271; Fig. 2G).

About incremental prognostic value analysis, Table 2 shows diabetes mellitus, FAI[LAD] ≥ −70.1, FAI[LCX] ≥ 70.1 and CT-FFR ≤ 0.80 were independent predictors of MACE in females according to univariate Cox analysis. Multivariate Cox proportional hazard modeling was performed to assess the prognostic value of FAI[LAD]/[LCX] over clinical predictors and CT-FFR in females (Table 3). Adding FAI[LCX] to the risk prediction model of CT-FFR and clinical risk factors provided incremental prognostic value (Harrel’s C 0.669, *P* < 0.001). However, a model with FAI[LAD] did not significantly predict outcomes better than model 1 (Harrel’s C 0.643, *P* = 0.054). The HR and *P* values of FAI[LAD] and FAI[LCX] were as follows: FAI[LAD] ≥ 70.1 (HR, 2.101; 95% CI, 1.055–4.182; *P* = 0.035), FAI[LCX] ≥ 70.1 (HR, 3.230; 95% CI, 1.982–5.265; *P* = 0.000), as shown in Table 3.

**Table 2:** Univariate Cox regression analysis for MACE in females and males

	Males		Females	
	HR (95% CI)	*P* value	HR (95% CI)	*P* value
Age	1.011 (0.995–1.028)	0.185	1.003 (0.980–1.028)	0.778
BMI	0.963 (0.912–1.016)	0.170	0.985 (0.929–1.043)	0.596
Hypertension	1.132 (0.776–1.651)	0.517	1.376 (0.879–2.156)	0.163
Diabetes mellitus	1.680 (1.140–2.476)	0.009	1.584 (1.050–2.389)	0.028
Hypercholesterolemia	0.953 (0.600-1.153)	0.837	1.080 (0.669–1.744)	0.753
Smoking history	1.031 (0.718–1.482)	0.867	1.806 (0.252–12.96)	0.557
Family history of CAD	0.049 (0.000-112.8)	0.445	0.049 (0.000-1102)	0.556
Aspirin/Clopidogrel	1.242 (0.698–2.208)	0.461	1.272 (0.694–2.334)	0.436
Beta-blocker	0.911 (0.636–1.303)	0.609	1.007 (0.668–1.518)	0.974
CCB	1.041 (0.709–1.530)	0.837	1.483 (0.947–2.322)	0.085
ACEI	0.891 (0.479–1.656)	0.715	1.523 (0.847–2.738)	0.160
Statin	1.367 (0.715–2.612)	0.344	1.714 (0.696–4.222)	0.241
FAI[LAD] ≥ −70.1	1.251 (0.673–2.325)	0.480	2.282 (1.147–4.541)	0.019
FAI[LCX] ≥ −70.1	1.054 (0.646–1.720)	0.834	3.382 (2.078–5.505)	0.000
FAI[RCA] ≥ −70.1	0.948 (0.442–2.035)	0.892	1.932 (0.935–3.990)	0.075
CT-FFR ≤ 0.80	2.533 (1.737–3.695)	0.000	2.560 (1.699–3.857)	0.000

HR, hazard ratios; BMI, body mass index; CAD, coronary artery disease; CCB, Calcium channel blocker; ACEI, angiotensin-converting enzyme inhibitor; FAI, fat attenuation index; LAD, left anterior descending artery; LCX, left circumflex; RCA; right coronary artery; CT-FFR, CT-derived fractional flow reserve

**Table 3:** Multivariable-adjusted Cox models for MACE in females

	HR (95% CI)	*P* value	Global Chi-square	C-statistic	C-statistic *P* value	Continuous NRI	NRI *P* value	Absolute IDI	IDI *P* value
Clinical predictor + CT-FFR ≤ 0.80 **(model 1)**			25.79	0.633	-	-	-	-	-
Diabetes mellitus	1.517 (1.005–2.289)	0.047							
CT-FFR ≤ 0.80	2.512 (1.667–3.786)	0.000							
model 1 + FAI[LAD] ≥ 70.1 **(model 2)**			30.72	0.643	0.054 (vs. model 1)	0.041 (-0.141-0.187)	0.189 (vs. model 1)	0.005 (-0.002-0.031)	0.259 (vs. model 1)
Diabetes mellitus	1.502 (0.995–2.267)	0.053							
CT-FFR ≤ 0.80	2.486 (1.649–3.748)	0.000							
FAI[LAD] ≥ −70.1	2.101 (1.055–4.182)	0.035							
model 1 + FAI[LCX] ≥ 70.1 **(model 3)**			52.08	0.669	< 0.001 (vs. model 1)	0.161 (0.073–0.260)	< 0.001 (vs. model 1)	0.036 (0.008–0.090)	= 0.010 (vs. model 1)
Diabetes mellitus	1.461 (0.968–2.207)	0.071							
CT-FFR ≤ 0.80	2.442 (1.619–3.683)	0.000							
FAI[LCX] ≥ −70.1	3.230 (1.982–5.265)	0.000							

MACE, major adverse cardiac events; HR, hazard ratios; NRI, net reclassification index; IDI, integrated discriminative improvement; CT-FFR, CT-derived fractional flow reserve; FAI fat attenuation index; LAD, left anterior descending; LCX, left circumflex

### Patient reclassification

Continuous NRI and absolute IDI were used to examine the ability of FAI[LAD]/[LCX] to appropriately reclassify patients at risk for MACE (Table 3). Added FAI[LAD] to model 1 didn’t show positive continuous NRI (0.041, *P* = 0.189) and absolute IDI (0.005, *P* = 0.259). The addition of FAI[LCX] to model 1 had a significant incremental reclassification effect over CT-FFR and clinical predictors with NRI of 0.161 (0.073–0.260), *P* < 0.001. Such increase in reclassification effect was also confirmed by absolute IDI (0.036; 95% CI 0.008–0.090, *P* = 0.010).

### Decision curve analysis

**Fig. 3:**
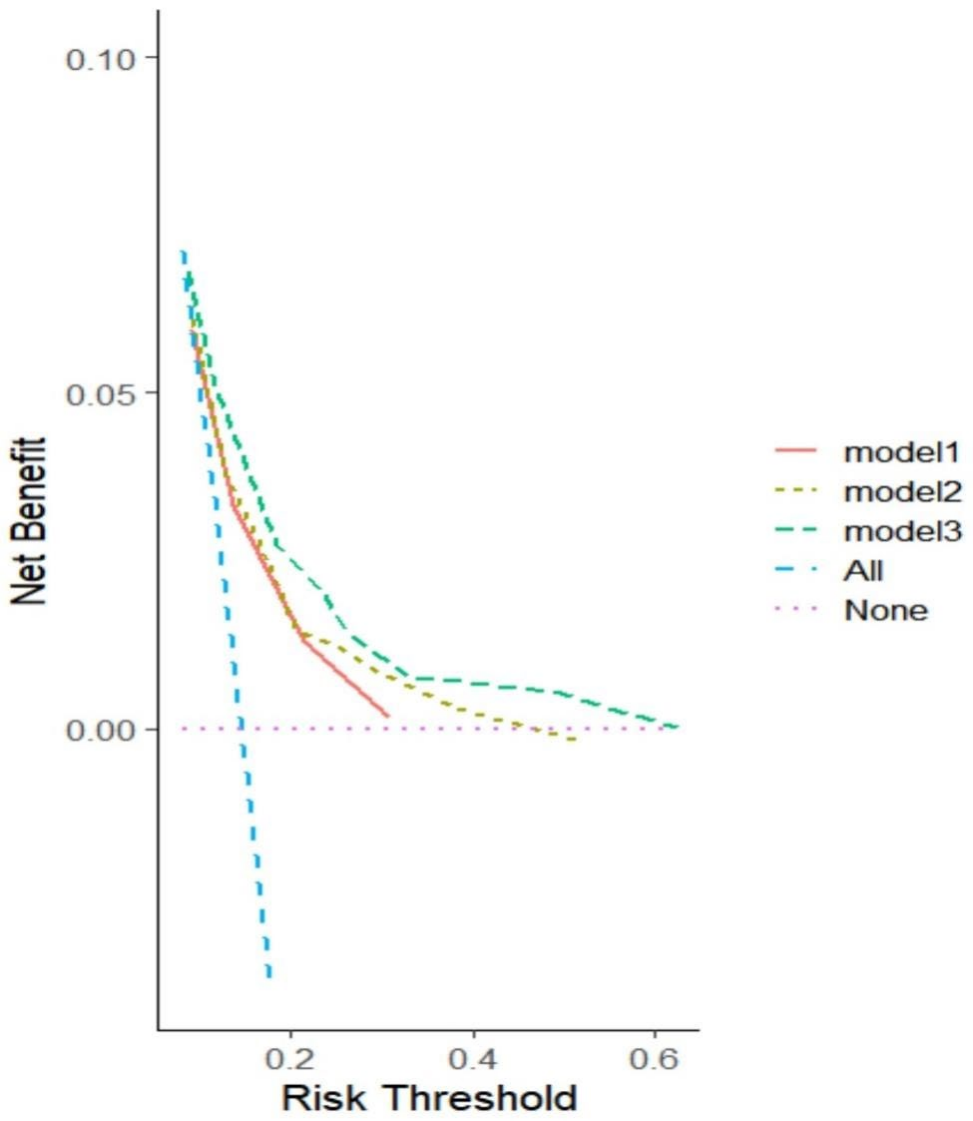
Decision curves analysis for the three models for predicting MACE in females. Model 1: Diabetes mellitus + CT-FFR ≤ 0.80; Model 2: model 1 + FAI[LAD] ≥ −70.1; Model 3: model 1 + FAI[LCX] ≥ −70.1. MACE, major adverse cardiac events; CT-FFR, CT-derived fractional flow reserve; FAI, fat attenuation index; LAD, left anterior descending; LCX, left circumflex

At decision curve analysis, model 3 provided the highest net benefit compared with the other two models (Fig. 3).

## Discussion

Our study is the first to evaluate the prognostic value of CT-FFR and FAI in patients with suspected CAD disaggregated by sex, and to investigate the incremental prognostic value of FAI over CT-FFR in both sex. From this study it emerged that CT-FFR ≤ 0.80 was associated with an increased rate of MACE in both sex. FAI[LAD] and FAI[LCX] ≥ 70.1 were associated with increased rate of MACE in females, and FAI[LCX] ≥ 70.1 provided incremental prognostic value over CT-FFR in females. We found a significant interaction between sex and FAI, but not with CT-FFR. FAI is likely females-specific risk factor, which highlights the need to consider differences in sex when applying FAI to predict MACE.

CT-FFR could predict MACE in CAD or suspected CAD as investigated in prior studies. According to ADVANCE trials, patients with CT-FFR > 0.8 had a significantly lower risk for MACE than patients with CT-FFR ≤ 0.8, suggesting that CT-FFR predicts MACE in these patients [17]. Another study showed that CT-FFR contributed enough to predict MACE in patients with acute coronary syndrome [18]. In addition, previous studies emphasized sex differences in coronary stenoses and found that females had significantly smaller coronary artery diameters which might affect CT-FFR calculation [9]. However, Stefan et al. observed that CT-FFR performed equally in men and women in diagnostic performance. A similar trend was observed in our study that the prognostic value of CT-FFR was not significantly biased by sex. Thus, for both sex in patients with suspected CAD, the use of CT-FFR was warranted to reduce the occurrence of MACE.

FAI is a novel parameter reflecting vessel inflammation [5]. Inflammation plays a pivotal role throughout all atherogenesis steps and is associated with MACE [5]. Oikonomou et al. demonstrated that FAI[LAD] and FAI[RCA] were associated with all-cause and cardiac mortality, whereas FAI[LCX] was associated with all-cause but not cardiac mortality [7]. Another study showed that RCA pericoronary adipose tissue computed tomography attenuation (PCATa) was associated with occurrence of death and nonfatal MI [19]. Susan et al. also suggested that FAI[RCA] > − 70.1 was a significant predictor of MACE (cardiac death, non-fatal myocardial infarction, hospitalization for any cardiac reason, and late revascularization during follow-up), and further reported that sex differences impact the prognostic value of FAI[RCA] [10]. When sexes were compared in clinical studies, plaque size was the most common measurement. However, most evidence suggested that plaque size did not correlate with plaque rupture in humans. Rather, plaque inflammation and morphology were better surrogates for plaque vulnerability, ischemic events, and mortality [20]. An exaggerated inflammatory response drives adverse vascular remodeling and that female sex hormones attenuate this to procure protection. However, menopause has a negative impact on vascular inflammation and endothelial function. Menopausal women with established CAD express greater levels of proinflammatory cytokines and associated with inflammation (raised hsCRP) and vascular dysfunction (reduced endothelium-dependent vasodilation) [21]. Plaque inflammatory cells through the damaged endothelium of the vessel into the subintima where they [22] become lipid-laden foam cells. Foam cells in the plaque secrete matrix metalloproteinases that degrade the fibrous cap overlying the plaque [23] and eventually result in plaque rupture, thrombosis, and acute ischemia-the cause of most MIs and strokes [24]. In our study, the age of females was older than males, and most of females were in the stage of menopause. Although, females showed significantly lower FAI[LAD]/[LCX]/[RCA] than males. This environment with less estrogen might make plaques more prone to rupture and increase the risk of adverse events. The above reasons might explain that FAI provided prognostic value in females but not in males. Of note, however, we did not observe a significant prognostic value of FAI[RCA] and the correlation between sex bias and FAI[RCA]. We found that FAI[LAD] and FAI[LCX] were associated with the increased rate of MACE in females, but not in males. Differences in inclusion criteria, different study endpoints, different follow-up times, the fact that all patients were Asian and the fact that the prognostic value of FAI seems to depend on the presence and localization of culprit lesions may account for the divergent finding.

In patients with suspected CAD, combining coronary flow velocity reserve and calcium score could improve the risk stratification ability and identify high-risk patients who could benefit from proper treatment [25]. Naya et al. demonstrated that direct coronary function measures might be more powerful marker of cardiac risk than simple assessment of the total atherosclerosis burden [26]. Nowadays, functional testing parameters of CT-FFR and FAI with noninvasive modality have played an important role in the diagnosis of CAD. However, only few study focus on the incremental prognostic value of FAI beyond CT-FFR. A previous study demonstrated the added prognostic value of FAI over fractional flow reserve (FFR) [27]. Susan et al. reported that FAI did not provide incremental prognostic value over myocardial perfusion imaging/CCTA including calcium scoring [10]. As far as we know, this is the first study to assess the added prognostic value of FAI over CT-FFR. In our study, FAI[LAD], FAI[LCX], and FAI[RCA] were not independent predictors of MACE in males, so these variables did not offer added prognostic value. In females, FAI[LAD] and FAI[LCX] had independent prognostic value. Adding FAI[LAD] ≥ 70.1 in model 1 did not improve C-index, the NRI and IDI. Therefore, FAI[LAD] ≥ 70.1 didn’t provide enhanced prediction value. However, FAI[LCX] ≥ 70.1 provided enhanced prediction and reclassified in females.

The added prognostic value of FAI[RCA] has previously been substantiated by vanDiemn et al. who observed RCA PCATa retained its prognostic value beyond quantitative plaque volume, high-risk plaques, and MI [19]. Contrary to the study, we found the added prognostic value of FAI[LCX] over CT-FFR, whereas FAI[LAD] and FAI[RCA] were not. A possible explanation for the discordancy is that the enhanced prognostic value of FAI appears to depend on the presence and localization of the culprit lesion, while in our experiment, a higher frequency of culprit lesions may present and locate in the LCX. These was illustrated by a study in which FAI_PVAT_ was increased around ruptured coronary atherosclerotic plaques of patients [5], and in our study, the value of FAI[LCX] was higher compared to FAI[LAD] and FAI[RCA]. Another possible explanation for this discordancy is the different definition of MACE. FAI of the RCA and LAD were associated with all-cause and cardiac mortality, whereas FAI[LCX] was associated with all-cause but not cardiac mortality [7]. In our study the definition of MACE was all-cause death, nonfatal MI, unplanned revascularization (more than 90 days after CCTA) and stroke. The different prognostic value of FAI might be linked with the different composition of MACE. However, further studies are needed to validate our hypothesis.

Our findings have potential clinical implications. First, these biomarkers can be obtained from CCTA images without additional scanning. Second, we focus on a unique cohort of suspected CAD patients disaggregated by sex, our findings showed that FAI was likely females-specific risk factor that was not routinely utilized in cardiovascular risk assessments. Third, our findings show the importance of activated inflammation in females, highlighting the importance of considering the enhanced prognostic value of FAI in females. The biomarker may represent potential treatment targets for MACE in females with CAD. Finally, FAI[LCX] not only increased the discriminative power of multivariate models including CT-FFR but also improved decision curve analysis within a practical range of threshold probabilities in females, suggesting that the measurement of FAI[LCX] may help to providing incremental risk stratification in female suspected CAD patients.

### Limitations

Our study possesses several limitations. First, selection and sampling bias was possible introduced because of the retrospective nature, the cohort consists of young patients, the use of a single-center database, and all patients were Asian. Therefore, the statistical power of our analysis may be limited, and results should be interpreted with caution. Second, the follow-up period of our study was relatively short. Therefore, longer periods of follow-up will be necessary. Finally, our study lacks some clinical data, such as low density lipoprotein, which will be investigated in further experiments.

## Conclusions

In suspected CAD patients, the prognostic value of CT-FFR is not significantly biased by sex. The prognostic value of FAI[LAD] and FAI[LCX] was significantly associated with MACE in females, but not males. FAI[LCX], not FAI[LAD], added incremental prognostic value over CT-FFR and might enhance CT-FFR risk stratification in females.

## Data Availability

The datasets generated during and/or analyzed during the current study are available from the corresponding author on reasonable request.

## Abbreviations

CAD: coronary artery disease
CT-FFR: CT-derived fractional flow reserve
FAI: fat attenuation index
CCTA: coronary computed tomographic angiography
MACE: major adverse cardiovascular events
LAD: left anterior descending
LCX: left circumflex
HR: hazard ratios
CI: confidence intervals
NRI: net reclassification improvement
IDI: integrated discrimination index
MI: myocardial infarction
RCA: the right coronary artery
SD: standard deviation
ACEI: angiotensin converting enzyme inhibitors
PCATa: pericoronary adipose tissue computed tomography attenuation
BMI: body mass index
CCB: calcium channel blocker
PDA: posterior descending artery
